# Fatty acid profile of muscles and adipose tissues of fat-tail Barbarine lambs as affected by rosemary residue intake

**DOI:** 10.5194/aab-63-431-2020

**Published:** 2020-11-28

**Authors:** Yathreb Yagoubi, Samir Smeti, Ilyes Mekki, Juan Ramón Bertolín, Guillermo Ripoll, Margalida Joy, Mokhtar Mahouachi, Naziha Atti

**Affiliations:** 1 University of Carthage, INRA-Tunisia, Animal and Forage Productions Laboratory, 2049 Ariana, Tunisia; 2 Centro de Investigacion y Technologia Agroalimentaria (CITA) de Aragón – IA2, Universidad de Zaragoza, Avda. Montañana, 930, 50059 Zaragoza, Spain; 3 Ecole Supérieure d'agriculture du Kef, University of Jendouba, Le Kef, Tunisia

## Abstract

The rosemary distillation industry produces a considerable amount of rosemary distillation residues (RRs), which can be an alternative for feeding animals in harsh conditions and could enhance animal product quality. Given the meat quality is largely influenced by its fat content and fatty acid composition, the fatty acid (FA) profiles of longissimus thoracis et lumborum (LTL), semi-membranous (SM) muscles, and caudal (CFs) and omental fats (OFs) were determined using 21 Barbarine lambs fed with or without RRs. Diets contained 600 g of concentrate plus 600 g of forage. Forage represented oat hay, RR87 and RR60 pellets containing 87 % or 60 % of RR, respectively. At the end of the study, all lambs were slaughtered, and the fatty acid profile was studied.

The inclusion of RR increased the polyunsaturated fatty acid (PUFA) contents and reduced saturated fatty acids (SFAs), and the thrombogenic and saturation indexes in all tissues. The SM muscle was the richest tissue in PUFAs, n-3 and n-6; however, both adipose tissues contained the highest proportions of SFAs. Especially the OF was the richest tissue in oleic acid and SFAs. Feeding RR to lambs enhanced meat quality.

## 1 Introduction

In recent years, the awareness of the importance of food in human health is widely increasing in the world. Consumers seek out natural and healthy meat, and they prefer to limit intake of saturated fatty acid (SFAs) and polyunsaturated FA (PUFAs). Ruminant meat differs from that of monogastric animals and contains a high SFA level and low PUFA : SFA ratio as a consequence of the ruminal biohydrogenation of forage and concentrate PUFAs into SFAs by rumen bacteria (French et al., 2000).

Many factors such as diet and breed could strongly affect muscle FA composition (Lobón et al., 2017). The use of nutritional strategies represents a beneficial approach to improve meat FA composition by using different PUFA-rich feed ingredients, such as linseed and algae (Atti et al., 2013; Hopkins et al., 2014). However, an excessive fat unsaturation may compromise meat oxidative stability, given its susceptibility to oxidation. Then, using dietary antioxidants is recommended (Lobón et al., 2017; Hopkins et al., 2014). As a natural dietary strategy, the residues of aromatic and medicinal plants, naturally rich in antioxidants, could play this role. Among these, rosemary is one of the most aromatic plants used in the distillation industry in the Mediterranean area. It covers a large area estimated to be 346 000 ha in Tunisia (Saadani, 2010), and its distillation industry generated about 5460 T yr${}^{-1}$ of residues. These rosemary residues (RRs) can be an efficient alternative feed that may totally or partially replace roughage and concentrate without altering animal performance and meat quality (Yagoubi et al., 2018). The effects of rosemary and thyme essential oils or by-products as additives on lamb meat quality has been studied (Nieto et al., 2010; Nieto, 2013; Smeti et al., 2018; Tibaoui et al., 2020), but their use as a basal ration for lambs is not very widespread.

On the other hand, sheep accumulate an important level of fat in different depots as mesenteric, omental, perirenal and caudal fat due to their ability to adapt to periods of food shortage (Atti et al., 2001; Wood et al., 2008). As an old tradition in the Middle East and northern Africa, lamb fat depots are used in various dishes and meat products (Silva et al., 2014). Therefore their FA profile determination becomes important for human health, given tissues and organs are known to have varying FA composition (Juárez et al., 2008), which reflect their differing physiological roles and metabolisms (Bell, 1979); therefore, their response to any dietary supply such as the RR with concentrate should be different. Hence, the objective of the current study was to evaluate the fatty acid profile in two adipose tissues and in two intramuscular lipids for Barbarine lambs as affected by an RR-based diet.

## 2 Material and methods

### 2.1 Animals and treatments

Twenty-one fat-tailed Barbarine lambs (body weight (BW): 23.7 kg) were randomly allocated to one of three basal diets. All diets contained 600 g of concentrate plus 600 g of forage. Forage was oat hay for the control group (C); pellets with 60 % of RR, 32 % of wheat bran and 8 % of soybean for RR60 group; and pellets containing 87 % of RR and 13 % of wheat bran for RR87 group. Chemical composition and FA profile of experimental diets are reported in Table 1.

**Table 1:** Chemical compositions (% dry matter) and fatty acid profiles as % FAMEs (mg FAME per 100 mg FAMEs) of experimental feeds.

	Concentrate	Oat hay	RR60	RR87
Dry matter	90.6	83.3	90.1	92.1
Crude protein	14.1	5.0	13.6	8.8
Crude fat	0.94	1.2	3.53	3.99
TPC	2.6	8.1	33.8	44.7
Fatty acid profile				
C14:0	0.37	3.16	0.94	1.62
C16:0	32.62	31.06	21.81	24.43
C16:1n-7	0.14	1.08	0.62	1.03
C17:0	0.2	1.24	0.37	0.5
C18:0	6.48	9.58	4.2	5.24
C18:1n-9	32.81	21.16	20.81	20.01
C18:2n-6	24.5	18.61	38.08	29.21
C18:3n-3	1.33	4.30	8.81	11.65
SFA	41.16	49.45	29.73	35.15
MUFA	33.24	23.88	21.62	21.2
PUFA	25.56	24.58	47.63	42.12
n-6PUFA	24.5	18.61	38.08	29.21
n-3PUFA	1.41	5.97	9.55	12.91
n-6 / n-3	17.4	3.11	3.98	2.26

TPC: total phenolic compound; SFA: saturated fatty acid; MUFA: monounsaturated fatty acid; PUFA: polyunsaturated fatty acid; RR60: rosemary pellets containing 60 % of rosemary residues (RRs), 32 % of wheat bran and 8 % of soybean; RR87: rosemary pellets containing 87 % of RR and 13 % of wheat bran.

### 2.2 Slaughter procedures and meat sampling

At the end of the experimental period (77 d), all lambs were fasted for 12 h with free access to water; then, they were weighed and slaughtered; the slaughter BW was 33 and 36 kg for C and both RR groups, respectively. After slaughter, a sample of omental fat was preserved. Then, the carcasses were chilled at 4 ${}^{\circ }$C for 24 h. The fat tails were then removed, and a sample of caudal fat from each animal was preserved. The left carcass halves were cut; the longissimus thoracis et lumborum (LTL) and semi-membranous (SM) muscles of each carcass were removed. Finally, all samples were frozen until FA profile determination.

### 2.3 Fatty acid analyses

The FA profile in all tissues was determined following the Lee et al. (2012) method and was expressed by % FAMEs (mg FAME per 100 mg FAMEs). For this, 0.4–0.8 g of lyophilized and minced samples were mixed with 1 mL of the internal standard (methyl tricosanoate, C23:0) and 2 mL of heptanes, and then 4 mL of NaOH / CH${}_{3}$OH (0.5 M) was added. The mixture was homogenized with vortex and heated for 20 min at 50 ${}^{\circ }$C. After cooling for 6–7 min, 4 mL of acetyl chloride / CH3OH (1/10 v/v) was added. The mixture was shaken and heated again for 60 min at 50 ${}^{\circ }$C. After cooling at ambient temperature, 2 mL of water Milli-Q was added; after homogenization, the mixture was shaken and centrifuged for 5 min, 3500 rpm at 10 ${}^{\circ }$C. The upper layer (heptanes) was extracted and transferred to a tube of 5 mL, and then the dehydration was performed with anhydrous Na${}_{2}$SO${}_{4}$. The mixture was shaken with vortex (30 s) and then centrifuged for 5 min (1000 rpm, 10 ${}^{\circ }$C); 1 mL of the supernatant was carefully transferred into a screw cap glass vial for gas chromatography. FAs were quantified as methyl ester (FAMEs). FAMEs were determined using a gas chromatograph (Bruker 436 Scion software Empower (GC)) equipped with 100 m × 0.25 mm D.I × 0.20 µm film thickness and a capillary column (BR-2560 Bruker) for the separation of fatty acids. The injector and detector were maintained at a temperature of 260 and 250 ${}^{\circ }$C, respectively. The injection volume was 1.0 µL, and the split ratio was 1 : 25. The FAME identification was based on retention times of fatty acid patterns of several reference methyl esters: GLC-463 and GLC-538. The desirable fatty acids were calculated as desirable fatty acid (DFA) = MUFA + PUFA + C18:0. The saturation (SI) and thrombogenic indexes (TIs) were calculated according to Ulbricht and Southgate (1991) as

$$\begin{array}{rl} \text{SI}= & (\text{C14:0}+\text{C16:0}+\text{C18:0})/\sum \text{MUFA}+\text{PUFA}\\ \text{TI}= & (\text{C14:0}+\text{C16:0}+\text{C18:0})/[0.5x\sum \text{MUFA}+0.5x\\ & /\sum (\text{n-6})+3x\sum (\text{n-3})+\sum (\text{n-3})/\sum (\text{n-6})]. \end{array}$$

### 2.4 Statistical analysis

Experimental data were analyzed by conducting an analysis of variance (ANOVA) to test the effect of an RR-based diet on a FA profile of LTL and SM muscles and omental and caudal fats using the general linear model (GLM) procedure of the SAS Institute (2004). The differences between groups were compared with the Duncan's multiple range test (DMRT). The significance was declared at p<0.05.

The following contrasts were used to compare the effects of the different diets:

- C1: the RR presence effect (C vs. RR60+RR87) and
- C2: the RR inclusion rate effect (RR60 vs. RR87). However, this contrast was tested and was not significant, for that it was not mentioned in tables.

Furthermore, to compare the fatty acid profile of all tissue types (muscles and adipose tissue), the following contrasts were calculated:

- C3: muscles vs. fats (M vs. F),
- C4: LTL vs. SM (LTL vs. SM) and
- C5: omental fat vs. caudal fat (OM vs. CF).

## 3 Results and discussion

As expected, the main FAs were palmitic (C16:0), stearic (C18:0) and oleic (C18:1) for all groups and all sites (muscles and adipose tissues). They accounted for 70 %–80 % of the total FA. These results were consistent with values commonly accepted for fat-tailed (Majdoub-Mathlouthi et al., 2015; Mekki et al., 2016) and thin-tailed sheep (Joy et al., 2012; Hopkins et al., 2014).

**Table 2:** Fatty acid profiles as % FAMEs (mg FAME per 100 mg FAMEs) of semi-membranous (SM) muscle from Barbarine lambs fed distilled rosemary residues.

	C	RR87	RR60	SEM	P	C1
Total fat	12.74	8.77	8.64	0.78	0.08	0.02
C10:0	0.15${}^{\text{a}}$	0.11${}^{\text{b}}$	0.14${}^{\text{a}}$	0.006	0.05	0.12
C12:0	0.08	0.05	0.07	0.008	0.46	0.47
C14:0	1.95	1.43	1.77	0.10	0.14	0.12
C15:0	0.26${}^{\text{b}}$	0.26${}^{\text{b}}$	0.40${}^{\text{a}}$	0.01	0.006	0.08
C16:0	24.66${}^{\text{a}}$	21.48${}^{\text{b}}$	22.94${}^{\text{ab}}$	0.41	0.01	0.01
C17:0	0.92${}^{\text{b}}$	1.23${}^{\text{b}}$	1.68${}^{\text{a}}$	0.08	0.007	0.009
C18:0	17.73${}^{\text{a}}$	14.91${}^{\text{b}}$	15.65${}^{\text{b}}$	0.27	0.001	0.001
C16:1 cis 9	1.83${}^{\text{a}}$	1.36${}^{\text{b}}$	1.43${}^{\text{ab}}$	0.08	0.08	0.02
C17:1 cis 10	0.82	1.08	1.09	0.05	0.07	0.02
C18:1 trans 11	0.64${}^{\text{b}}$	1.89${}^{\text{a}}$	1.58${}^{\text{a}}$	0.14	0.005	0.002
C18:1 cis 9	38.69	36.56	35.25	0.72	0.18	0.08
C18:2 n6	4.70${}^{\text{b}}$	9.19${}^{\text{a}}$	8.13${}^{\text{a}}$	0.39	0.001	0.001
C18:3 n3	0.25${}^{\text{b}}$	0.58${}^{\text{a}}$	0.55${}^{\text{a}}$	0.02	0.001	0.001
C18:2 cis 9,trans 11 (CLA)	0.30	0.34	0.36	0.01	0.45	0.23
C18:2 trans 9,trans 11) CLA	0.06${}^{\text{b}}$	0.13${}^{\text{a}}$	0.10${}^{\text{a}}$	0.006	0.002	0.001
C20:3 n9	0.47	0.41	0.33	0.03	0.36	0.22
C20:3 n6	0.16	0.23	0.23	0.01	0.08	0.03
C20:4 n6	2.0${}^{\text{b}}$	3.27${}^{\text{a}}$	2.92${}^{\text{ab}}$	0.19	0.03	0.01
C20:5 n3	0.10${}^{\text{b}}$	0.21${}^{\text{a}}$	0.21${}^{\text{a}}$	0.01	0.009	0.002
C22:4 n6	0.15${}^{\text{b}}$	0.29${}^{\text{a}}$	0.28${}^{\text{a}}$	0.01	0.001	0.001
C22:5 n3	0.29${}^{\text{b}}$	0.57${}^{\text{a}}$	0.60${}^{\text{a}}$	0.03	0.002	0.001
SFA	48.95${}^{\text{a}}$	43.12${}^{\text{c}}$	46.14${}^{\text{b}}$	0.38	0.001	0.001
MUFA	42.29	41.25	39.71	0.79	0.42	0.29
PUFA	8.74${}^{\text{b}}$	15.61${}^{\text{a}}$	14.15${}^{\text{a}}$	0.68	0.001	0.001
PUFA / SFA	0.17${}^{\text{b}}$	0.36${}^{\text{a}}$	0.30${}^{\text{a}}$	0.01	0.001	0.001
n-6PUFA	7.14${}^{\text{b}}$	13.19${}^{\text{a}}$	11.79${}^{\text{a}}$	0.60	0.001	0.001
n-3PUFA	0.71${}^{\text{b}}$	1.49${}^{\text{a}}$	1.51${}^{\text{a}}$	0.07	0.001	0.001
n-6 / n-3	9.86${}^{\text{a}}$	8.95${}^{\text{ab}}$	8.01${}^{\text{b}}$	0.28	0.05	0.03
CLA	0.39	0.51	0.50	0.02	0.11	0.03
DFA	68.8${}^{\text{b}}$	71.8${}^{\text{a}}$	69.5${}^{\text{b}}$	0.40	0.01	0.04
Saturation index	0.87${}^{\text{a}}$	0.66${}^{\text{c}}$	0.75${}^{\text{b}}$	0.01	0.001	0.001
Thrombogenic index	6.28${}^{\text{a}}$	1.19${}^{\text{b}}$	1.32${}^{\text{b}}$	0.82	0.03	0.01

SFA: saturated fatty acid; MUFA: monounsaturated fatty acid; PUFA: polyunsaturated fatty acid; CLA: conjugated linoleic acid; DFA: desirable fatty acid; saturation index = (C14:0 + C16:0 + C18:0) / ∑MUFA + PUFA; thrombogenic index = (C14:0 + C16:0 + C18:0) / [0.5x∑MUFA + 0.5x / ∑(n-6) + 3x∑(n-3) + ∑(n-3) / ∑(n-6)];C1: C vs. RR60 + RR87;a, b, c: values within a row different superscripts differ significantly at P< 0.05.

**Table 3:** Fatty acid profiles as % FAMEs (mg FAME per 100 mg FAMEs) of longissimus thoracis et lumborum (LTL) muscle from Barbarine lambs fed distilled rosemary residues.

	C	RR87	RR60	SEM	P	C1
Total fat	15.6	16.96	14.96	1.44	0.84	0.9
C16:0	24.99	23.39	25.01	0.34	0.11	0.29
C18:0	19.81${}^{\text{a}}$	17.72${}^{\text{ab}}$	16.74${}^{\text{b}}$	0.46	0.04	0.01
C16:1 cis 9	1.88	1.76	1.87	0.08	0.79	0.70
C18:1 cis 9	39.38	39.37	38.32	0.53	0.65	0.64
C18:2 n6	3.53${}^{\text{b}}$	5.09${}^{\text{a}}$	4.80${}^{\text{a}}$	0.24	0.04	0.01
C18:3 n3	0.24${}^{\text{b}}$	0.50${}^{\text{a}}$	0.45${}^{\text{a}}$	0.01	0.001	0.001
SFA	50.57${}^{\text{a}}$	47.11${}^{\text{b}}$	48.71${}^{\text{ab}}$	0.47	0.02	0.01
MUFA	43.1	43.4	45.0	0.51	0.29	0.32
PUFA	6.33	7.88	7.88	0.36	0.16	0.06
PUFA / SFA	0.12	0.16	0.16	0.006	0.10	0.03
n-6PUFA	5.10	6.46	6.39	0.33	0.20	0.07
n-3PUFA	0.56${}^{\text{b}}$	0.80${}^{\text{a}}$	0.85${}^{\text{a}}$	0.03	0.004	0.001
n-6 / n-3	9.1${}^{\text{a}}$	8.1${}^{\text{ab}}$	7.5${}^{\text{b}}$	0.27	0.10	0.04
CLA	0.40	0.47	0.47	0.01	0.29	0.12
DFA	69.2${}^{\text{ab}}$	70.6${}^{\text{a}}$	68.0${}^{\text{b}}$	0.33	0.02	0.91
Saturation index	0.95${}^{\text{a}}$	0.81${}^{\text{b}}$	0.86${}^{\text{b}}$	0.01	0.01	0.006
Thrombogenic index	1.8${}^{\text{a}}$	1.53${}^{\text{b}}$	1.6${}^{\text{b}}$	0.03	0.006	0.002

SFA: saturated fatty acid; PUFA: polyunsaturated fatty acid; CLA: conjugated linoleic acid; DFA: desirable fatty acid; saturation index = (C14:0 + C16:0 + C18:0) / ∑MUFA + PUFA;thrombogenic index = (C14:0 + C16:0 + C18:0) / [0.5x∑MUFA + 0.5x / ∑(n-6) + 3x∑(n-3) + ∑(n-3) / ∑(n-6)];C1: C vs. RR60 + RR87;a, b: values within a row different superscripts differ significantly at P< 0.05

### 3.1 Fatty acid composition of muscles (semi-membranous (SM) and longissimus thoracis et lumborum (LTL))

The determination of FA profile in SM muscle revealed 49 FA isomers. Palmitic and stearic acids were reduced with RR-based diets compared to the control (Table 2). However, oleic acid proportion was not altered (P>0.05). Proportion of margaric acid (C17:0) increased with RR intake compared to the control group. This result is consistent with Smeti et al. (2018), who recorded higher C17:0 proportions with rosemary essential oil intake. The trans-vaccenic acid (C18:1 trans 11) was higher in both RR diets, which can be considered a desirable result given its beneficial effect on human health. The RR intake did not affect the total conjugated linoleic acid (CLA) proportion, this being a value within the range of values reported earlier in lambs fed different diets (Leticia et al., 2017). For long-chain FAs, the higher proportions were observed for RR groups in ARA (20:4 n6), EPA (C20:5 n3), C22:4 n6 and DPA (C22:5 n3). The highest content of SFA was recorded for the C group and MUFA proportion was unaffected by dietary treatments, whereas proportions of total PUFA, n-6 and n-3 were significantly enhanced by RR intake. The abundance of linoleic and linolenic acids in SM muscle resulted from the richness of RR diets in these FAs (Wood et al., 2008). The PUFA / SFA ratio from SM muscle was enhanced with RR-based diets, but it was lower than the ratio recommended for a healthy diet (Department of Health, 1994). The TI for both RR groups was 25 % lower than that of the C group. This result is in line with previous results (Nieto, 2013; Smeti et al., 2018) that reported a significant decrease of the thrombogenic index using essential oils and by-products of rosemary and thyme in sheep feeding. The TIs recorded with RR60 and RR87 diets (1.25) are around the appropriate value (1) for a healthy diet (Sinanoglou et al., 2013). These values were comparable to those reported by Oriani et al. (2005) in Merino lambs (1.35). The SI index showed significant differences (P< 0.05); in agreement with Nieto (2013), meat from RR60 and RR87 had lower values than that of the C. Hence, we could conclude that the inclusion of rosemary residues in a lamb's diet can contribute to the prevention of cardiovascular diseases, diabetes and cancer (Nieto, 2013). The higher proportions of DFA observed in SM muscle from lambs receiving the high proportion of RR (71.8 for RR87 vs. 68.8 % for the Control) suggest that feeding lambs with rosemary by-products could increase the proportion of salubrious FA in produced meat.

For LTL muscle, a total of 49 FAs were recorded. The RR intake affected the SFA content; the highest (P< 0.05) proportion was observed in the control group (Table 3). Palmitic and oleic acid contents were similar among groups; however, stearic acid proportion was reduced with RR regimens (P< 0.05). MUFA content was similar for all groups (P> 0.05) and averaged 43.8 %; this is the consequence of similar MUFA intake. Total PUFA and n-6PUFA were unaffected by dietary treatments, while n-3PUFA increased with RR intake mainly due to the contribution of linolenic acid (C18:3n-3). The n-6 / n-3 ratio was significantly reduced in both RR diets, although it was still above the recommended value for human nutrition. Conversely, Leticia et al. (2017), studying the use of sage distillation by-products, did not find a clear effect on this ratio. In the present study, the RR intake decreased (P< 0.05) the SI (0.83 for RR vs. 0.95 for C). Similar results were recorded in meat lambs when the thyme by-products were included in pregnant and lactating ewes' diets (Nieto, 2013). The TI was slightly higher for the C group. This result agrees with the recent results of Smeti et al. (2018) using rosemary essential oil in lamb diets. More details of this muscle were mentioned in Yagoubi et al. (2018).

**Table 4:** Fatty acid profiles as % FAMEs (mg FAME per 100 mg FAMEs) of caudal fat (CF) from Barbarine lambs fed distilled rosemary residues.

	C	RR87	RR60	SEM	P	C1
C10:0	0.18	0.20	0.23	0.01	0.48	0.32
C12:0	0.12	0.09	0.11	0.008	0.53	0.38
C14:0	3.05	2.88	3.18	0.16	0.78	0.95
C15:0	1.01${}^{\text{b}}$	1.27${}^{\text{b}}$	1.75${}^{\text{a}}$	0.07	0.001	0.003
C16:0	22.54	23.11	22.98	0.52	0.89	0.65
C17:0	2.72${}^{\text{b}}$	3.22${}^{\text{ab}}$	3.56${}^{\text{a}}$	0.12	0.03	0.01
C18:0	16.62${}^{\text{a}}$	12.23${}^{\text{b}}$	11.43${}^{\text{b}}$	0.68	0.01	0.004
C20:0	0.12	0.09	0.09	0.006	0.11	0.04
C16:1 cis 9	3.18${}^{\text{a}}$	2.56${}^{\text{b}}$	2.95${}^{\text{a}}$	0.07	0.009	0.01
C17:1 cis 10	1.45${}^{\text{b}}$	1.88${}^{\text{ab}}$	1.98${}^{\text{a}}$	0.09	0.08	0.02
C18:1 trans 11	2.20${}^{\text{b}}$	4.25${}^{\text{a}}$	3.55${}^{\text{a}}$	0.25	0.01	0.006
C18:1 cis 9	37.5${}^{\text{a}}$	36.1${}^{\text{a}}$	32.9${}^{\text{b}}$	0.64	0.01	0.02
C18:2 n6	2.17${}^{\text{b}}$	4.15${}^{\text{a}}$	3.91${}^{\text{a}}$	0.21	0.002	0.001
C18:3 n3	0.29${}^{\text{b}}$	0.57${}^{\text{a}}$	0.59${}^{\text{a}}$	0.02	0.001	0.001
C18:2 cis 9,	0.60	0.57	0.69	0.03	0.37	0.75
trans 11 (CLA)						
C20:4 n6	0.10	0.14	0.14	0.006	0.18	0.06
SFA	48.4	45.2	46.6	0.86	0.34	0.18
MUFA	45.3	45.5	42.4	0.62	0.10	0.31
PUFA	3.55${}^{\text{b}}$	5.83${}^{\text{a}}$	5.74${}^{\text{a}}$	0.25	0.002	0.001
PUFA / SFA	0.07${}^{\text{b}}$	0.13${}^{\text{a}}$	0.12${}^{\text{a}}$	0.006	0.009	0.002
n-6PUFA	2.46${}^{\text{b}}$	4.46${}^{\text{a}}$	4.25${}^{\text{a}}$	0.22	0.003	0.001
n-3PUFA	0.36${}^{\text{b}}$	0.66${}^{\text{a}}$	0.67${}^{\text{a}}$	0.03	0.001	0.001
n-6 / n-3	6.94	6.77	6.37	0.31	0.74	0.58
CLA	0.68	0.67	0.79	0.03	0.41	0.57
DFA	65.5${}^{\text{a}}$	63.5${}^{\text{a}}$	59.6${}^{\text{b}}$	0.74	0.01	0.02
Saturation index	0.87	0.75	0.78	0.03	0.30	0.13
Thrombogenic	1.69	1.43	1.48	0.05	0.19	0.07
index						

SFA: saturated fatty acid; MUFA: monounsaturated fatty acid; PUFA: polyunsaturated fatty acid; CLA: conjugated linoleic acid; DFA: desirable fatty acid; saturation index = (C14:0 + C16:0 + C18:0) / ∑MUFA + PUFA; thrombogenic index = (C14:0 + C16:0 + C18:0) / [0.5x∑MUFA + 0.5x / ∑(n-6) + 3x∑(n-3) + ∑(n-3) / ∑(n-6)];C1: C vs. RR60 + RR87;a, b: values within a row different superscripts differ significantly at P< 0.05.

### 3.2 Fatty acid composition of adipose tissues (caudal fat (CF)and omental fat (OF))

The determination of FA profile in CF revealed 55 FAs. The stearic acid and oleic acid were significantly affected by the lamb's diet (P< 0.01; Table 4), whereas the palmitic acid was unaffected (P> 0.05). The C15:0 and the C17:0 proportions increased only in RR60 (P< 0.05). The inclusion of rosemary residue in the diet, regardless of the amount, increased the trans-vaccenic acid (C18:1 trans 11). The contents of SFA and MUFA groups were not affected by the diet (P> 0.05), with average contents of 46.73 % and 44.4 %, respectively. However, both RR diets resulted in higher (P< 0.05) C18:2n-6 and C18:3n-3. The proportions of linoleic acid are in the range (1.8 %–12.9 %) of a previous study (Santos-Silva et al., 2003). The PUFA content and the PUFA / SFA ratio were affected by the diet (P< 0.01), being higher in both RR treatments; however, this ratio is still lower than the recommended value (0.45). This result is in relationship to the low PUFA value and the high SFA level (Sinclair, 2007). The n-6 and n-3PUFA were enhanced (P< 0.05) for RR60 and RR87 groups; these higher proportions, mainly due to linoleic and linolenic acids, could result from differences in dietary intake of these FA. The rosemary residues are rich in linoleic and linolenic, the precursors of n-3 and n-6 series of FA (Velasco et al., 2001). The higher proportions of linoleic and linolenic acids may be protective against cardiovascular diseases (Scollan et al., 2001). The n-6 / n-3 ratio was unaffected by the diet (P> 0.05), and the average value was always above the recommended value. The proportion of DFAs that include all unsaturated FAs and stearic acid in caudal fat ranged from 59.6 % to 65.5 %. This proportion was higher for C and RR87 than RR60; however, it is within the range (61 %–80 %) reported for other red-meat animal species (Banskalieva et al., 2000). The total CLA proportion, the saturation and thrombogenic indexes were unaffected by dietary treatments (P> 0.05).

**Table 5:** Fatty acid profiles as % FAMEs (mg FAME per 100 mg FAMEs) of omental fat (OF) from Barbarine lambs fed distilled rosemary residues.

	C	RR87	RR60	SEM	P	C1
C10:0	0.17	0.17	0.20	0.006	0.30	0.59
C12:0	0.10	0.10	0.12	0.006	0.45	0.52
C14:0	2.70	2.66	3.04	0.10	0.28	0.50
C15:0	0.71${}^{\text{b}}$	0.71${}^{\text{b}}$	0.94${}^{\text{a}}$	0.02	0.002	0.04
C16:0	23.3${}^{\text{a}}$	21.4${}^{\text{b}}$	22.4${}^{\text{ab}}$	0.31	0.07	0.05
C17:0	2.18${}^{\text{b}}$	2.75${}^{\text{ab}}$	3.32${}^{\text{a}}$	0.11	0.003	0.002
C18:0	29.14${}^{\text{a}}$	23.4${}^{\text{b}}$	22.9${}^{\text{b}}$	0.55	0.001	0.001
C20:0	0.21${}^{\text{a}}$	0.14${}^{\text{b}}$	0.13${}^{\text{b}}$	0.008	0.005	0.001
C16:1 cis 9	2.56${}^{\text{a}}$	2.04${}^{\text{b}}$	2.15${}^{\text{b}}$	0.03	0.001	0.001
C17:1 cis 10	0.70${}^{\text{b}}$	0.88${}^{\text{ab}}$	1.03${}^{\text{a}}$	0.04	0.03	0.02
C18:1 trans 11	2.49${}^{\text{b}}$	5.66${}^{\text{a}}$	4.70${}^{\text{a}}$	0.25	0.003	0.001
C18:1 cis 9	29.97	31.5	30.1	0.47	0.37	0.41
C18:2 n6	0.03	0.02	0.02	0.002	0.15	0.07
trans 9, 12						
C18:2 n6	2.45${}^{\text{b}}$	4.81${}^{\text{a}}$	4.93${}^{\text{a}}$	0.21	0.003	0.001
C18:3 n3	0.24${}^{\text{b}}$	0.67${}^{\text{a}}$	0.74${}^{\text{a}}$	0.02	0.001	0.001
C18:2 cis 9,	0.34	0.40	0.44	0.01	0.13	0.06
trans 11 CLA						
C20:4 n6	0.05${}^{\text{b}}$	0.12${}^{\text{a}}$	0.10${}^{\text{ab}}$	0.008	0.02	0.01
SFA	59.7${}^{\text{a}}$	52.6${}^{\text{b}}$	54.4${}^{\text{b}}$	0.74	0.002	0.0007
MUFA	36.6${}^{\text{b}}$	40.8${}^{\text{a}}$	36.6${}^{\text{b}}$	0.59	0.02	0.02
PUFA	3.38${}^{\text{b}}$	6.41${}^{\text{a}}$	6.58${}^{\text{a}}$	0.24	0.001	0.001
PUFA / SFA	0.06${}^{\text{b}}$	0.12${}^{\text{a}}$	0.12${}^{\text{a}}$	0.004	0.001	0.001
n-6PUFA	2.61${}^{\text{b}}$	5.08${}^{\text{a}}$	5.16${}^{\text{a}}$	0.22	0.001	0.001
n-3PUFA	0.31${}^{\text{b}}$	0.76${}^{\text{a}}$	0.82${}^{\text{a}}$	0.02	0.001	0.001
n-6 / n-3	8.41${}^{\text{a}}$	6.58${}^{\text{b}}$	6.27${}^{\text{b}}$	0.17	0.001	0.001
CLA	0.41${}^{\text{b}}$	0.54${}^{\text{a}}$	0.57${}^{\text{a}}$	0.01	0.01	0.003
DFA	69.1${}^{\text{ab}}$	70.6${}^{\text{a}}$	68.2${}^{\text{b}}$	1.67	0.008	0.66
Saturation index	1.39${}^{\text{a}}$	1.01${}^{\text{b}}$	1.07${}^{\text{b}}$	0.03	0.001	0.001
Thrombogenic	2.71${}^{\text{a}}$	1.89${}^{\text{b}}$	1.98${}^{\text{b}}$	0.33	0.001	0.001
index						

SFA: saturated fatty acid; MUFA: monounsaturated fatty acid; PUFA: polyunsaturated fatty acid; CLA: conjugated linoleic acid; DFA: desirable fatty acid; saturation index = (C14:0 + C16:0 + C18:0) / ∑MUFA + PUFA; thrombogenic index = (C14:0 + C16:0 + C18:0) / [0.5x∑MUFA + 0.5x / ∑(n-6) + 3x∑(n-3) + ∑(n-3) / ∑(n-6)];C1: C vs. RR60 + RR87;a, b: values within a row different superscripts differ significantly at P< 0.05.

**Table 6:** Fatty acid profiles as % FAMEs (mg FAME per 100 mg FAMEs) of four tissues from Barbarine lambs.

	Muscle (M)		Fat (F)		Contrasts				
	LTL	SM	OF	CF	C3	C4	C5	P	SEM
< C14:0	0.25${}^{\text{c}}$	0.22${}^{\text{c}}$	0.36${}^{\text{b}}$	0.84${}^{\text{a}}$	0.001	0.62	0.001	0.001	0.02
C14:0	2.09${}^{\text{b}}$	1.71${}^{\text{c}}$	2.80${}^{\text{a}}$	3.04${}^{\text{a}}$	0.001	0.03	0.16	0.001	0.06
∑C15:0	0.58${}^{\text{c}}$	0.46${}^{\text{c}}$	1.29${}^{\text{b}}$	2.15${}^{\text{a}}$	0.001	0.28	0.001	0.001	0.03
C16:0	24.5${}^{\text{a}}$	23.02${}^{\text{b}}$	22.4${}^{\text{b}}$	22.9${}^{\text{b}}$	0.01	0.02	0.42	0.007	0.21
C16:1 cis 9	1.84${}^{\text{c}}$	1.54${}^{\text{d}}$	2.25${}^{\text{b}}$	2.89${}^{\text{a}}$	0.001	0.01	0.001	0.001	0.04
C17:0	1.62${}^{\text{c}}$	1.28${}^{\text{c}}$	2.75${}^{\text{b}}$	3.16${}^{\text{a}}$	0.001	0.08	0.03	0.001	0.06
C17:1 cis 10	0.94${}^{\text{b}}$	1.00${}^{\text{b}}$	0.87${}^{\text{b}}$	1.77${}^{\text{a}}$	0.001	0.56	0.001	0.001	0.03
C18:0	18.1${}^{\text{b}}$	16.1${}^{\text{c}}$	25.17${}^{\text{a}}$	13.42${}^{\text{d}}$	0.001	0.03	0.001	0.001	0.33
C18:1 cis 9	39.02${}^{\text{a}}$	36.83${}^{\text{b}}$	30.53${}^{\text{c}}$	35.48${}^{\text{b}}$	0.001	0.01	0.001	0.001	0.31
C18:2n6	4.47${}^{\text{b}}$	7.34${}^{\text{a}}$	4.06${}^{\text{b}}$	3.41${}^{\text{b}}$	0.001	0.001	0.23	0.001	0.19
C18:3 n3	0.4${}^{\text{b}}$	0.46${}^{\text{ab}}$	0.55${}^{\text{a}}$	0.48${}^{\text{ab}}$	0.05	0.28	0.28	0.11	0.02
C20:0	0.08${}^{\text{b}}$	0.08${}^{\text{b}}$	0.16${}^{\text{a}}$	0.10${}^{\text{b}}$	0.001	0.85	0.001	0.001	0.004
C20:3n9	0.19${}^{\text{b}}$	0.40${}^{\text{a}}$	0.05${}^{\text{c}}$	0.05${}^{\text{c}}$	0.001	0.001	0.94	0.001	0.009
C20:3n6	0.09${}^{\text{b}}$	0.20${}^{\text{a}}$	0.02${}^{\text{c}}$	0.02${}^{\text{c}}$	0.001	0.001	0.98	0.001	0.004
C20:4n6	1.19${}^{\text{b}}$	2.73${}^{\text{a}}$	0.09${}^{\text{c}}$	0.12${}^{\text{c}}$	0.001	0.001	0.84	0.001	0.05
C20:5n3	0.08${}^{\text{b}}$	0.17${}^{\text{a}}$	0.01${}^{\text{c}}$	0.01${}^{\text{c}}$	0.001	0.001	0.91	0.001	0.004
C22:4n6	0.10${}^{\text{b}}$	0.24${}^{\text{a}}$	0.01${}^{\text{c}}$	0.02${}^{\text{c}}$	0.001	0.001	0.88	0.001	0.005
C22:5n3	0.21${}^{\text{b}}$	0.49${}^{\text{a}}$	0.05${}^{\text{c}}$	0.05${}^{\text{c}}$	0.001	0.001	0.99	0.001	0.01
C22:6n3	0.03${}^{\text{b}}$	0.08${}^{\text{a}}$	0.009${}^{\text{c}}$	0.007${}^{\text{c}}$	0.001	0.001	0.77	0.001	0.002
Even-chain SFA	45.01${}^{\text{b}}$	41.20${}^{\text{c}}$	50.85${}^{\text{a}}$	39.80${}^{\text{c}}$	0.01	0.003	0.001	0.001	0.44
Odd-chain SFA	2.04${}^{\text{c}}$	1.61${}^{\text{c}}$	3.59${}^{\text{b}}$	4.63${}^{\text{a}}$	0.001	0.10	0.001	0.001	0.09
SFA	48.8${}^{\text{b}}$	46.1${}^{\text{c}}$	55.7${}^{\text{a}}$	46.7${}^{\text{b}}$	0.001	0.01	0.001	0.001	0.39
PUFA	7.36${}^{\text{b}}$	12.83${}^{\text{a}}$	5.46${}^{\text{c}}$	5.04${}^{\text{c}}$	0.001	0.001	0.60	0.001	0.28
PUFA / SFA	0.15${}^{\text{b}}$	0.28${}^{\text{a}}$	0.16${}^{\text{b}}$	0.11${}^{\text{b}}$	0.01	0.004	0.25	0.001	0.01
n-6	5.98${}^{\text{b}}$	10.7${}^{\text{a}}$	4.28${}^{\text{c}}$	3.72${}^{\text{c}}$	0.001	0.001	0.42	0.001	0.24
n-3	0.73${}^{\text{b}}$	1.24${}^{\text{a}}$	0.76${}^{\text{b}}$	0.57${}^{\text{b}}$	0.001	0.001	0.17	0.001	0.04
n-6 / n-3	8.2${}^{\text{a}}$	8.94${}^{\text{a}}$	6.93${}^{\text{b}}$	6.69${}^{\text{b}}$	0.001	0.11	0.61	0.001	0.19
CLA	0.45	0.47	0.67	0.71	0.01	0.86	0.70	0.08	0.04

C3: muscles vs. fats (M vs. F); C4: longissimus thoracis et lumborum (LTL) vs. semi-membranous (SM); C5: omental fat vs. caudal fat (OM vs. CF);even-chain SFA: ∑C10:0; C12:0; C14:0; C16:0; C18:0; C20:0; C22:0; C24:0;odd-chain SFA∑C13:0; C15:0; C17:0; C23:0;SFA: saturated fatty acid; PUFA: polyunsaturated fatty acid; CLA: conjugated linoleic acid;a, b, c: values within a row different superscripts differ significantly at P< 0.05.

For omental fat, a total of 55 FAs were recorded. As in the CF, the palmitic and stearic acids were significantly affected by the lamb's diet and were higher for the C group than both RR diets (Table 5). However, the oleic acid was similar among groups (P> 0.05). Nevertheless, the omental depot contained more stearic acid and less oleic acid than the caudal fat. As for the other studied tissues, the C17:0, the linoleic and linolenic acids, C17:1 cis 10 and C18:1 trans 11 were significantly higher (P< 0.05) for RR-based diets. However, C16:1 cis 9 was higher for the control diet. The SFA proportion was significantly higher in the C group than both RR-based diets. This high content of SFA is explained by the biohydrogenation process in rumen of unsaturated FA into SFA. Similar results in omental fat were found for SFA content, which averaged 59.24 % in lambs fed different levels of spineless cactus (Costa et al., 2017). The RR87 presented the highest proportion (P< 0.05) of MUFAs compared to the other treatments. The MUFAs have been associated with a reduced cardiovascular disease risk as they decrease plasma low-density lipoprotein (LDL) concentration (Costa et al., 2017). Among the MUFAs, the oleic acid represents the higher proportion for all groups without a significant difference. Oleic acid is considered resistant to oxidative processes in foods and increases the shelf life of certain products (Scollan et al., 2001). The highest amount of PUFAs was made up of linoleic acid (C18:2n-6), which was doubled for RR-based diets (P< 0.05). The RR regimens resulted in an increase of PUFA proportion considered important for human cells and for the performance of essential functions in the human body. As in CF, the PUFA : SFA ratio was significantly affected by the diet being higher for both RRs (P< 0.05). The higher n-6 and n-3PUFA proportions (P< 0.05) for RR60 and RR87 are mainly due to the higher linoleic and linolenic acids proportions. These FAs are considered to be essential FAs given they cannot be synthesized by the body (Wood et al., 2008). Similar results in sheep fed spineless cactus presenting an average of 0.79 % of n-3PUFA were reported (Costa et al., 2017). The n-6 / n-3 ratio was reduced with RR intake (6.58 and 6.27 vs. 8.41 for RR87, RR60 and C, respectively), but it is still above the recommended value (4) for all regimens. The total CLA was slightly higher for RR diets. The saturation and thrombogenic indexes were higher for the C group than experimental ones.

### 3.3 Comparison of fatty acid profiles of four tissues

Regardless of the diet, the FA profile of the muscles differed significantly from that of the adipose tissues for the majority of individual FAs, groups and ratios (Table 6). It was reported that the FA composition depends on location of tissues in the carcass (Monziols et al., 2007). According to our study, the adipose tissues contained more individual SFAs (C14:0, ∑C15:0, C17:0) than muscles. However, LTL contains higher C16:0 than other tissues. The omental fat had the highest content of C18:0, while the caudal fat had the lowest content of this FA. Intermediate values were recorded for LTL and SM muscles. It was reported that adipose tissues are considered the major sites of de novo FA synthesis, palmitic acid being the main product, which can be further elongated to stearic acid (Sinclair, 2007).

Fat depots contained the higher proportions of short (< C14:0) odd- and even-chain SFA compared to muscles. These results are in line with those of Pellattiero et al. (2015). The even-chain SFA was significantly higher in OF (P< 0.05) than in other tissues; however the odd-chain SFA was lower in both muscle sites than in fat depots. The myristic acid (C14:0) contributed to a significantly higher proportion in adipose tissues than in muscles (2.9 % vs. 1.9 %). The difference among the FA composition of fat and muscles is pronounced mainly for the abundant FAs. In general, the LTL and SM muscles contained higher proportions of C18:1 cis 9 (39 % and 36.8 %, respectively) and higher C18:2n6 particularly for SM muscle (4.47 % and 7.34 %, respectively) and an intermediate value of C18:0 compared to omental and caudal fat (18.1 % and 16.1 % vs. 25.17 % and 13.42 % for LTL, SM, OF and CF, respectively). The C18:1 cis 9 was reduced in omental fat and increased in LTL tissue; however the values of SM and caudal fat tissues were similar and intermediate. The C18:1, as previously shown (Joy et al., 2012; Hopkins et al., 2014; Mekki et al., 2016), is the most abundant FA in all the studied depots, followed by palmitic acid and stearic acid. In the current study, the OF contained the highest proportion of total SFA (55.7 %, Table 6), mainly due to the higher level of stearic acid (C18:0), while the SM muscle contained the lowest SFA level (46.1 %) and the CF and LTL muscle had intermediate values.

The adipose tissues contained significantly lower total PUFA, n-3 and n-6PUFA than the muscles. The highest PUFA was recorded in SM muscle (Table 6); it is largely related to the increased amounts of total n-6, which was doubled in SM muscle resulting from higher content of C18:2n6. In addition, the total amount of n-3 was higher for SM than the rest of tissues. Some differences were observed among LTL and SM muscles. Then, SM muscle was the richest muscle in PUFA (12.8 %) mainly due to the contribution of C18:2n6 and FAs with chains longer than 18C including other fatty acids present in trace amounts in other tissues, among them C20:3n9, C20:3n6, C20:4n6ARA, C20:5n3EPA and C22:5n3DPA. These differences among muscles can be related to the presence of fibers with different oxidative or oxido-glycolytic properties. These fibers are present in various proportions in muscles depending on animal species, location of the muscle on the body and physiological characteristics such as physical movement. It was reported that red oxidative fibers have a higher proportion of phospholipids than white fibers and consequently a higher percentage of PUFAs (Pellattiero et al., 2015). The PUFA contents of the current study are higher than those previously reported (Enser et al., 1996) in ovine and bovine, which averaged 5.88 % and 4.86 %, respectively.

## 4 Conclusion

Feeding sheep with rosemary residues resulted in different fatty acid profiles that depend also on the tissue type. This study showed the existence of a saturation gradient between tissues subject to their location. Adipose tissues, especially the omental fat, contained the highest proportions of SFA; then, it is recommended to avoid the intake of internal and external fats, as it has been seen that they are rich in SFAs, which are undesirable for human health. However, they can be used, in low amounts, to formulate meat products to enhance their nutritional value.

## Data Availability

The original data of the paper are available upon request to the corresponding author.
